# Clicking for supper

**DOI:** 10.7554/eLife.07690

**Published:** 2015-04-29

**Authors:** Peter Tyack

**Affiliations:** School of Biology, University of St. Andrews, St. Andrews, United Kingdomplt@st-andrews.ac.uk

**Keywords:** *Phocoena phocoena*, echolocation, beam directionality, buzz, prey capture, convergent evolution, other

## Abstract

When close to prey, porpoises actively widen their sonar beam, which may make it harder for the prey to escape.

**Related research article** Wisniewska DM, Ratcliffe JM, Beedholm K, Christensen CB, Johnson M, Koblitz JC, Wahlberg M, Madsen PT. 2015. Range-dependent flexibility in the acoustic field of view of echolocating porpoises (*Phocoena phocoena*). *eLife* **4**:e05651. doi: 10.7554/eLife.05651

**Image** Porpoises change the shape of a fatty structure called the melon (yellow) to alter the width of their sonar beam

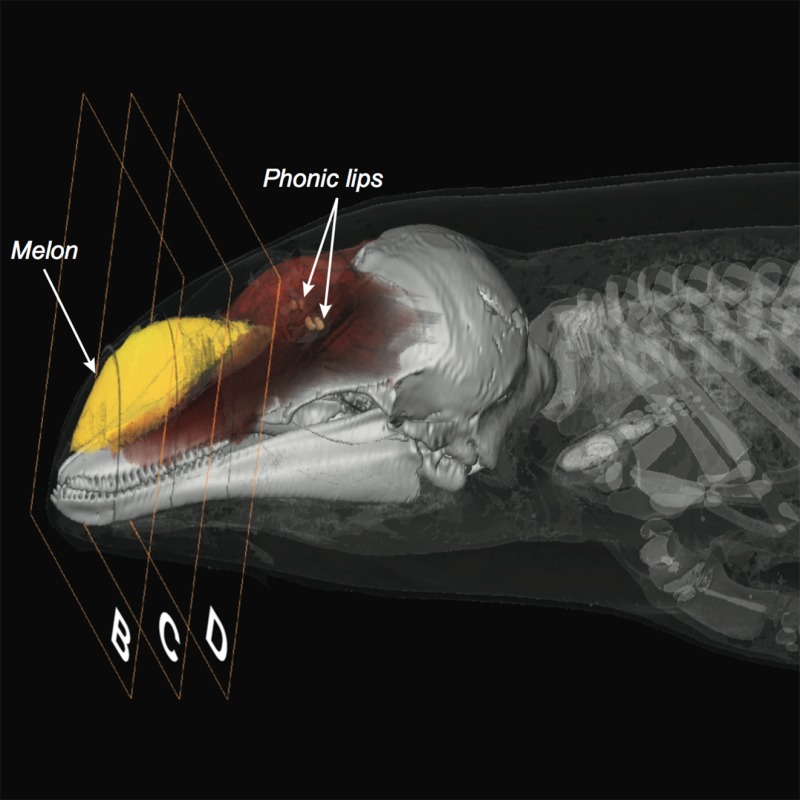

Animals use sonar to solve many problems; the most complicated of these is to find, select and capture prey. Early research on dolphin sonar focused on simpler artificial problems. During the Cold War, navies wanted to find better ways to detect submarines and mines so they funded much of this research, which made many surprising discoveries. For example, dolphins can hear frequencies that are 10 times higher than we can hear, and can detect a 1 inch metal sphere 100 meters away. Sperm whales can make sonar sounds as intense as a naval destroyer. However, although navies may care about using sonar to detect a metal object, a dolphin in the wild doesn't.

We are finally seeing research into how toothed whales (porpoises, dolphins, and larger whales such as the sperm whale with teeth instead of baleen) use sonar to solve their own problems, and this work reveals unsuspected flexibility and sophistication (Figure 1). Now, in *eLife*, Danuta Wisniewska of Aarhus University and colleagues show that porpoises can focus their sonar beam as they approach a fish, but then widen the beam, perhaps to keep it in ‘view’ even if it tries to escape (Wisniewska et al., 2015).

**Figure 1.**
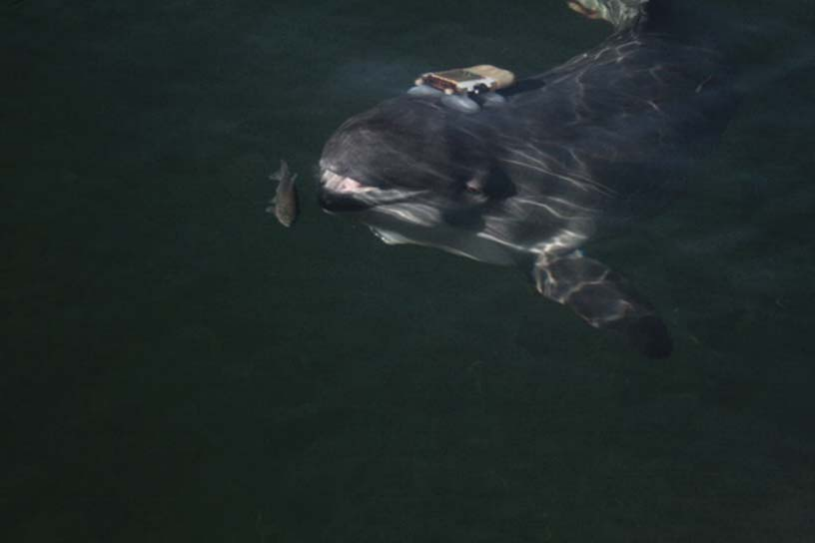
A porpoise using echolocation to find and capture a fish at the Fjord and Belt research facility where the research by Wisniewska et al. was conducted. The porpoise is wearing a tag that records the outgoing clicks and echoes returning from objects in this environment.

IMAGE CREDIT: MONIKA DYNDO/FJORD AND BELT.

As with companies advertising the power of their amplifiers, early papers on whale biosonar competed to report the loudest click from each species. But each whale makes clicks that vary in loudness over several orders of magnitude. In 2003, researchers started to make sense out of this variation when they reported that several species make loud clicks when far from a target, but reduce the loudness of their clicks as they approach (Au and Benoit-Bird, 2003). The researchers suggested that by making fainter clicks when it is close to a target, the dolphin stabilizes the loudness of the echo, making it easier for its auditory system to analyze. But one puzzle remained: none of the studied animals reduced the loudness enough to keep the echo at a constant level.

Human-built sonars also need to adapt their settings to detect objects at different distances, but the power of their transmitter seldom varies. Instead, the sensitivity of the receiver automatically increases for faraway targets. It took a Russian neurobiologist collaborating with an American lab to test whether dolphins change their hearing sensitivity as well as the loudness of their clicks. These researchers wondered how dolphins could protect their ears from their very loud outgoing clicks and then listen for faint echoes. It emerged that a dolphin reduces its hearing sensitivity when it clicks, and that the sensitivity then increases again over time in a way that makes the echo level more stable (reviewed in Supin and Nachtigall, 2012). But why change two things—the loudness of each click (the ‘transmit gain’) and the sensitivity of their hearing (the ‘receiver gain’)—when one mode of gain control could do?

Perhaps physiology constrains how much transmit or receiver gain can change with time. On the other hand, thinking ecologically, perhaps it is a hint that dolphins keep the level of their click constant at the target. Why would dolphins control sound level at the target rather than loudness of the echo as they hear it? Most fish are only able to hear low frequency sound, but some fish (such as herring) are able to hear high frequencies. Indeed, shad swim away from high frequency clicks, which could suggest that these fish evolved specialized hearing in order to escape echolocating predators (Wilson et al., 2011). When killer whales feed on prey that might be able to hear their echolocation clicks, they use more cryptic echolocation than when feeding on deafer prey. Perhaps dolphins also adjust the level of their clicks as they approach some prey to reduce the risk that prey may hear and avoid them. The way dolphins reduce click level to maintain a constant level at the prey would be just right for this problem, perhaps requiring them to perform the rest of the gain control by changing the sensitivity of their hearing.

The echolocation clicks of bats and whales are among the most intense biological sounds, but it is difficult for even these animals to produce sounds powerful enough to detect echoes at long range. A key to the sonar of bats and whales is that they direct sound in a narrow beam. In a dolphin, louder clicks have a higher center frequency and a narrower beam (Au et al., 1995). This narrowing of the beam for louder clicks could help to focus energy when an animal is struggling to detect a distant target. And Wisniewska et al. point out that as an echolocating predator closes on its prey, widening the sonar beam may make it harder for the prey to escape detection. Widening the beam will also dilute the sound energy reaching the prey, perhaps reducing the risk that the prey will detect the clicks.

Wisniewska et al.—who are based at Aarhus, the University of Southern Denmark, the University of St Andrews and the University of Tübingen—observed how porpoises change their sonar signals during different phases of catching prey (Wisniewska et al., 2015). These results help to make sense out of earlier observations that clicks reduce in level and frequency as a dolphin approaches a target, but the width of the sonar beam increases. Beam width was earlier thought to change as a function of click frequency based simply upon the physics of sound production, while Wisniewska et al. show that beam pattern is under voluntary control.

The most important result of Wisniewska et al. to me is their report that the porpoises were able to flexibly modify the beam pattern independently of the level and frequency of the clicks. An organ called the ‘melon’, which is near the forehead and contains specialized acoustic fats, was shown to change in shape as the porpoise changes the pattern of its sonar beam. Wisniewska et al. argue that highly innervated muscles surrounding the melon enable the dolphin to rapidly change the beam pattern under voluntary control. Earlier work has used CT scans and computers to model how the anatomy of the dolphin head forms the basic beam pattern in dolphins (Aroyan et al., 1992). A challenge for future work will be to model exactly how changes in three structures—the sound production organ, the air sacs in the upper respiratory tract, and the shape of the melon—actually modify the sonar beam of porpoises.

When we study how an animal solves its problems in real life situations, we can start to understand the complexity of its behavior. Future work would do well to apply the kind of careful acoustic analysis of sonar performed by Wisniewska et al. to ecological interactions between echolocating marine predators and their prey. How close does the predator have to be to detect the prey with echolocation? How far away does the prey need to be to hear and avoid the predator? How does the predator select which target it wants to eat? Once the predator attacks, how effectively can prey get out of the sonar beam? What are the counterstrategies of the predator? These are the kinds of observations that would help us to understand the sophistication with which toothed whales use different kinds of sonar signal to detect, select, approach and capture prey as opposed to detecting a metal object.
